# A meta-analysis approach to measure the impact of project-based learning outcome with program attainment on student learning using fuzzy inference systems

**DOI:** 10.1016/j.heliyon.2022.e10248

**Published:** 2022-08-13

**Authors:** Mukta Goyal, Chetna Gupta, Varun Gupta

**Affiliations:** a Jaypee Institute of Information Technology, Noida, 201305, India; b University of Alcala, Alcalá de Henares, Madrid, 28802, Spain; c School of Business, University of Applied Sciences and Arts Northwestern Switzerland, 4600 Olten, Switzerland

**Keywords:** Project-based learning, Bloom taxonomy, Course attainment parameters, Program attainment parameters, Fuzzy logic, Outcome based education, Assessment for learning

## Abstract

This paper presents a fuzzy inference method to investigate the impact of project-based assessment on the desirable outcomes by analyzing students creative and critical thinking, collaborative decision-making, and communication skills with realistic constraints and standards through theory and practical implementation in (a) course attainment and (b) on overall program attainment carried out in engineering discipline. This paper uses twelve specific parameters to capture program attainment parameters (PAPs). It proposes three main parameters to define various assessment system elements required for assessing course attainment parameters (CAPs), correlated with each other. To the best of the author's knowledge, to date, there is no defined mathematical tool to map CAPs to PAPs. Thus, this paper proposes assessment pedagogy to evaluate the PAPs corresponding to CAPs to handle the vague correlation mapping using fuzzy logic. The methodology and the preliminary results conducted for one year are promising, helping educators evaluate a candidate's performance individually or in a group on several assessment criteria, assisting in attaining the knowledge, values, attitude, deep learning, and skills needed for sustainable education development.

## 1 Introduction

With the advancement in education, there has been a paradigm shift in assessment techniques adapted to measure comprehensive knowledge and higher-order skills, namely creativity, innovation, critical thinking, coordination, and communication, problem-solving, etc. [1]. This shift has led to the emergence of the Assessment for Learning (AFL) movement, in which teaching, learning, and assessment are closely linked. To investigate the impact of learning, the assessment criteria's for a candidate should balance the need for deep understanding, integration of knowledge, application of prior knowledge with practical use alluding to the purpose, the ultimate goal of learning for achieving specific course, and program attainment [1]. Researchers have focused on the relevance of assessment in the past by emphasizing corrective assessment measures to teach higher-order thinking, the ability to solve problems, and decision making [2, 3, 4]. Assessment should be similar to what happens and evaluated in the professional field, including collaborative or peer-to-peer work. The structure, assessment criteria, and expectations of authentic assessment should be transparent and known in advance. More recently, educators are using more innovative ways to assess students' knowledge by redefining traditional assessment methods [1, 2, 3, 4]. In higher education, innovative assessment is a collaborative effort that recognizes personal perceptions and reactions to learning [4].

One of major challenge higher education face is to ensure the holistic development of student both in terms of attaining generic attributes and development of competencies, namely creativity, thinking, teamwork, communication and collaboration, independence. To address these challenges, new technological transformations in education has led to the use of additional instructional tools, such as project-based learning (PBL) [5, 6, 7], augmented reality (AR) [8], active learning [9], etc. to facilitate the achievement of such attributes and competencies. Educational goals of any institutes are based on Bloom's taxonomy that classifies into knowledge, skills, and attitudes [10]. Regarding higher studies, especially the engineering discipline, PBL is practiced right from the first year as an integral part of the curriculum and plays an essential role in demonstrating knowledge and understanding. It is the minimum set of skills to be processed by the graduating engineers, defined through the program attainment parameters (PAPs), and measured at graduation [11]. Technical universities have their Program Objectives (PO) and Program Educational Objectives (PEO) for the Bachelor of Engineering degree program. The projects could have many different solutions that are reached in various ways, making the outcomes vary from group to group. Assessment is a critical component of learning, and PBL assessment criteria are based on PAPs. The creation of rubrics, reflections, peer- and self-evaluations, and any other assessment tool must be carefully crafted. By assessing the different aspects of the project and project creation, the students have more opportunities to make up for an area they may not excel at. The PAPs are addressed through the outcome of the Course Attainment (CA) parameters. There is a correlation between PAPs and CAs for practical measures related to the skills, knowledge, and behavior in a particular course. In general, all the higher education curriculum courses have 4–7 course attainments, mapped to PAPs using a correlation mapping matrix on a scale of 1–3, where 1 means low correlation and 3 means high correlation. The final assessment is performed on criteria based on PAPs and indicates how well the learning is imparted. It also reflects how well the successful running graduate programs. A rubric-based in the course attainment parameter leads to more understanding and good quality of the project as rubrics are considered standard measurement tools to access the program outcomes' attainment. Using rubrics makes assessing students on the 4C's, creative/critical thinking, collaboration, communication, and creativity [12], much more simplistic and objective, which can be used to access both individual and group grades. It establishes the need to incorporate teaching methods equipped with sustainable development (ESD) skill sets and knowledge that the market demand. It will help universities make undergraduates fulfill the industry's requirements and increase the students' levels of interest toward design, problem-solving, and independent learning.

Thus, this paper investigates the project-based assessment outcome carried out in higher education, particularly in engineering. This paper examines the impact of knowledge gained, critical thinking development, and collaborative decision-making skills through theory and practical implementation on (a) course attainment and (b) on overall program attainment. The attainment outcome is measured in different dimensions of assessment criteria used by assessment developers, policymakers at the university level, and supervisors as they work to create and adopt assessments. It promotes more profound learning of 21st century skills, promoting more in-depth learning, values, and skills needed to succeed in today's knowledge-based economy, shaping a sustainable future through education [9]. To conduct this experiment, the data is collected systematically for a final year project for one year in two consecutive semesters in a controlled environment to access the fulfillment of learning skills required to include creative and critical thinking, collaboration, communication, creativity, realistic constraints, and standards. This work is based on twelve distinctive program attainment parameters (PAPs) and three main parameters to define various assessment system elements required for assessing course attainments (CAs), correlated with each other. Fuzzy logic, along with Mamdani Inference Method [13], is used to evaluate the PAPs corresponding to CAP's to handle the vague correlation mapping matrix between PAPs and CAs. The concept of Learning Analytics (LA) [14] is used to collect, analyze, measure, and report investigating data about learners' knowledge. The effectiveness of the proposed is depicted by the statistical analysis performed on the undergraduate students' evaluation results and performance. The main contributions of this research are as follows:

• The proposed PBL assessment framework contributes towards sustainability in higher education by investigating a successful initiative and its outcomes, helping educators assess a candidate's performance individually or in a group. It uses twelve distinctive parameters to capture program attainment parameters (PAPs) and three main parameters to define various assessment elements for assessing course attainments (CAs), correlated with each other. It helps in accessing the knowledge, values, attitude, deep-learning, and skills needed for sustainable development education.
• Mamdani Inference Method [13] is used to evaluate the PAPs corresponding to CAPs to handle the vague correlation mapping using the proposed mathematical tool.
• Step-by-step implementation of the PBL framework is presented, which can help attain the competencies required for ESD by analyzing the effects of PBL implementation for engineering undergraduate-level courses spanned in two consecutive semesters.
• Statistical results show significant evidence of different impacts of the variations on different categories of attainment in the course and program level.

The following research questions are investigated to demonstrate the achievement of desired outcomes:

RQ 1. Is there a direct relationship between the effectiveness of course learning and the performance of a student?
RQ 2. Does the student performance affect the course attainment parameter?
RQ 3. Does the mathematical tool establish the relation between the course attainment parameter (CAP1, CAP2, and CAP3) and program attainment parameter (PAP1, PAP2…PAP12)?

The remainder of the paper is organized as follows: Section 2 discusses the background and section 3 discusses the proposed fuzzy-based PBL assessment framework followed by results and the study's findings in section 4. Finally, the conclusion is presented in section 5.

## 2 Background

In the traditional lecture method of covering academic content, the learning rate was shallow. There is no doubt that the conventional lecturing method is still considered an effective teaching method, mainly in science, technology, engineering, and mathematics (STEM). Several authors have explored and recognized the effectiveness of project-based learning (PBL) in higher education (with a focus on engineering education) in different countries [15, 16, 17] and have applied the concept of PBL either through industry collaboration or standalone in their respective workplaces. For instance, Hasaan and et al. [18] adopted an integrated, multicourse, project-based learning methodology in electronic engineering in Spain. Ruikar and et al. [19] collaborated with the industry through multimedia podcasting in the UK. Another study activity theory is used to investigate the use of project-based learning in Ireland [20]. However, the results of this study were mixed due to some contradictions that were detected activity system. Some researchers have also adopted project-based learning in collaboration with students and teachers, claiming that student-teachers can become better problem-solvers together [21].

A study conducted by [22] on the effectiveness of incorporating PBL indicates that PBL is preferred among teachers. This study was conducted in primary schools and vocational secondary schools. Another study conducted by [23] presents a cross-course PBL approach (for requirement engineering, project management, and software engineering courses). It reports the summary results obtained from student evaluations assessed for eight years using various cross-course PBL efforts. Their findings show that their approach can be useful in Requirement Engineering, Project Management, and Software Engineering courses. The prominent advantages of integrating PBL in higher education include enhanced student motivation [24], learning various skills independently, or gaining in-depth understanding. It helps students integrate and develop collaboration and execution skills [25] and suitability for a wide range of students and learning styles [26]. The work presented in [27] shows the effectiveness of using wikis for PBL in three undergraduate courses of different disciplines, namely, English Language Studies, Information Management, and Mechanical Engineering. Their study concludes that students mostly hold positive attitudes towards the use of wikis for project-based learning.

Research has demonstrated how curriculum, assessment, and evaluation are based on which the program is built and acts as the primary tools to evaluate the quality of teaching and learning achieved through course and program attainment parameters [28]. Kaviet et al. [29] have explained the hierarchy of faculty involvement in CO-PO mapping and demonstrates how student learning can be empowered through CO-PO attainment. Attainment expected results in student learning, where attainment is the essential standard of academic attainment [29]. Assessment is broadly categorized into direct and indirect methods to access CO's and PO's. The former focuses on accessing student performance through acquired knowledge and skill, whereas the latter focuses on reflecting views on students learning based on surveys and interviews. Different stakeholder's opinions regarding graduate's knowledge and skills are collected by institutes [30]. Nakkeeran et al. [31], present the results of their findings by advocating that it is mandatory to shift from the traditional education system to Outcome-Based Education (OBE), including PO, PSO, and CO. In another work presented in [32] shows that how the PBL framework serves as an efficient pedagogy model to improve program outcome attainments using the PBL approach. Troussas et al. [33] proposes a fuzzy inference method for delivering language learning material in a dynamic manner. It's a hybrid model for detecting and identifying misconceptions, as well as an inference system for dynamically delivering learning objects matched to learners' needs via machine learning. Yang Tzu-Chi et al [34] presents the finding of their research on enhance students learning using a 2 × 2 factorial design. Their work intends to determine the impacts of the observational learning (OL) or Self-regulated learning on students' online learning performance.

## 3 Proposed research methodology

**Fig: 1:**
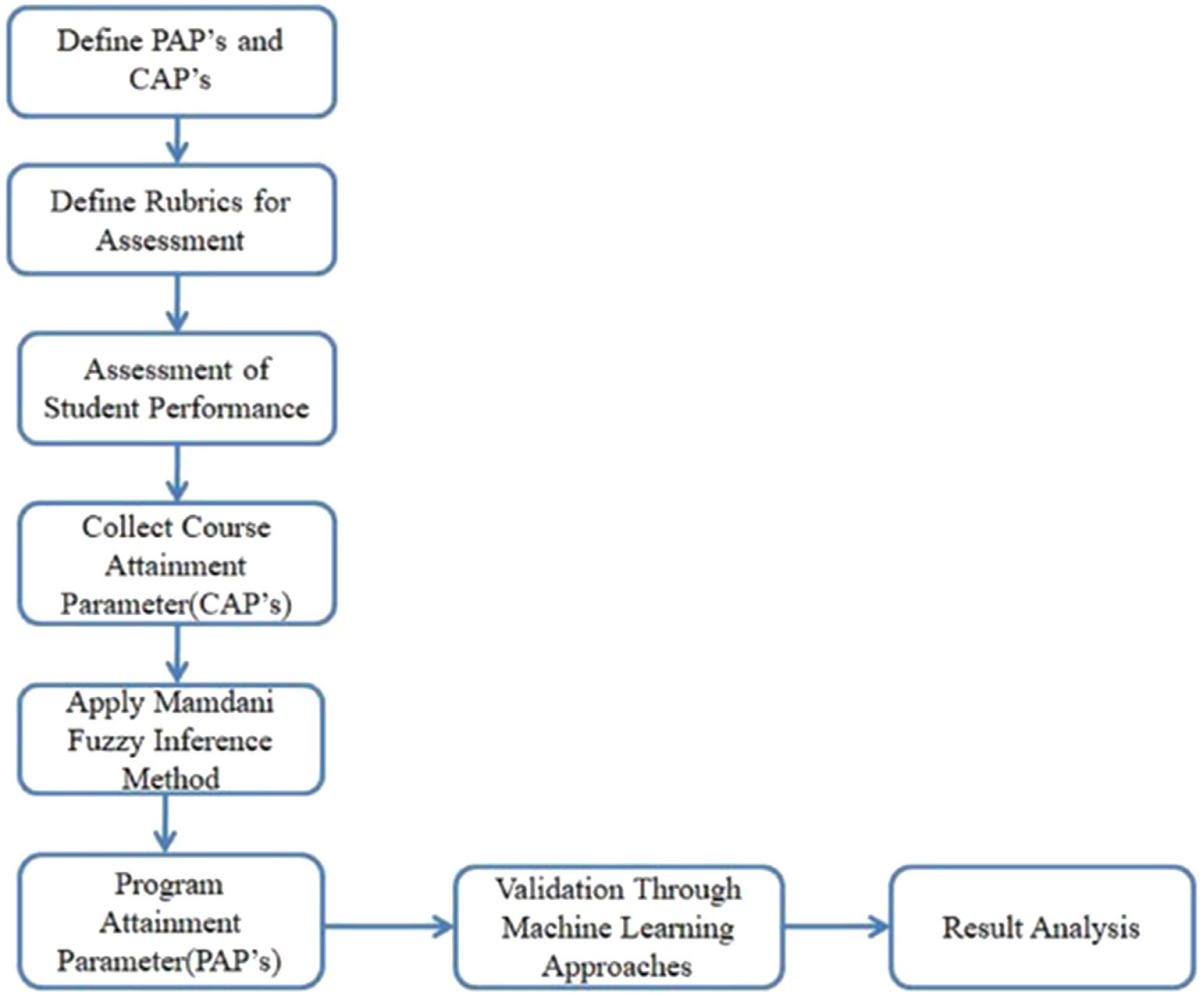
A proposed process model to calculate PAPs corresponding to CAPs.

The higher education system has its policies to determine the PAPs. Accreditation agencies such as ABET, NBA, NACC, etc. [35] defines Program Learning Objectives (PLO's)/Program Attainment Parameters (PAPs). It is an integral part of Science, Technology, Engineering, and Mathematics (STEM) where educational concepts are coupled with real-world lessons for better learning. More frequently, the average of the direct assessment and indirect assessment is considered for mapping it to the course [36]. The indirect assessment component is computed from the feedback of the students. The challenge here is to map the PAPs corresponding to CAPs. Currently, the course instructor uses their previous knowledge to map the PAPs corresponding to CAPs. Due to the uncertainty and vague nature of the human mind, a Mamdani Inference Method [13] is used to handle the course instructor's decision-making to map the PAPs. There is no defined mathematical tool to map these CAPs to PAPs to the best of our knowledge. Thus, this paper proposes an assessment pedagogy to evaluate the PAPs corresponding to CAPs to handle the vague correlation mapping. Figure 1 shows the process model to calculate the program attainment parameter corresponding to CAPs.

### 3.1 Proposed course attainment parameters

This paper proposes three-course attainment parameters and defines its various assessment system elements at the course attainment level. The proposed course attainment level and the mapping of Bloom's taxonomy (most widely used and accepted) with all twelve program attainment parameters discussed in [35] are listed in Table 1. These are considered for mapping with proposed course attainment parameters to fulfill the fulfillment of desired learning outcomes.

**Table 1:** Course Attainment Parameters and their mapping.

	Course Attainment Parameters (CAP)	Level	PAP Mapping
CAP 1	**Assessment of Intellectual Learning and Understanding:** a) It includes recalling of knowledge of key concepts, b) Facts and theories to build their critical, ethical, and reasonable thinking	Understand Level	PAP1, PAP2
CAP 2	**Assessment of Ability to Investigate and Apply:** a) it includes displaying analytical knowledge gained by investigating problem solutions through acquired knowledge and techniques in different ways and at varying abstraction levels. b) It also includes peer collaboration, planning, and modeling. c) Complex problem solving and research.	Analyze and Apply Level	PAP 2, PAP3, PAP4, PAP5, PAP6, PAP7, PAP8, PAP9
CAP 3	**Assessment of Validation and Acceptability of Different Alternatives:** a) To prepare a candidate for future-ready positions in both industry and academia, b) A rigorous assessment in the context of making and defending judgments about the applicability and validity of ideas and solutions for providing support in real life is a must.	Evaluate Level	PAP10, PAP 11, PAP12

### 3.2 Proposed rubric's assessment

A rubric is designed in Table 2 to achieve proposed CAPs. Literature suggests that there is no one fit-size model for quantifying performance. In PBL, the evaluators must be specific about their expectations from passing graduates as much as possible [35]. Creating a rubric for the same will indeed provide the expectation from every attainment. A broad guideline of distribution of marks to understand the project assurance for various project work components can be gained for more accurate, specific, and useful assessment. These program attainment parameters are mapped with the subject experts' course attainment parameters based on Bloom's taxonomy [10], having six cognitive domain levels, namely, *Remember, Understand, Apply, Analyze, Evaluate and Create*. The project evaluation strategy is based on this rubric to ensure a fair and unbiased evaluation of every project and every member. A program evaluation committee comprises three faculty members (for every assessment) to ensure uniformity and unbiasedness. Each parameter's final score is the weighted average calculated through pre-assigned program attainment rubrics (refer to Table 2). In the light of innovative assessment, the proposed approach will help educators assess a candidate's performance on several assessment criteria revolving around the mentioned core standards.

**Table 2:** Rubrics for Proposed PBL based Project Evaluation.

Parameters	Exemplary (≥80%)	Competent (≥50% &<80%)	Unsatisfactory (<50%)
Literature Survey	Referred to more than ten papers from a reputed journal. Study of tools and current techniques	Some of the documents from the conference and some from a reputable journal. No study of Tools	Paper studied from the conferences, not from a reputed journal.
Problem Identification and Formulation	A problem that is not implemented earlier and students are clear, how to proceed further.	Problem definition is clear but not feasible for implementation.	The problem is not defined clearly.
Design/Methodology	The proposed algorithm performance is better than the existing algorithm.	The proposed algorithm performance is similar to the existing algorithm.	No algorithm is proposed
Coding/Implementation	The Proposed algorithm is implemented using the current tools and technology	The working prototype of the project is implemented, but there are some issues.	The only front end is implemented. No backend
Result Analysis	Precise analysis of the result and comparative analysis with other techniques are performed.	Analysis of the result in an elaborated method, but does not compare with other techniques.	No result analysis
Viva Voice/Presentation	Knowledge of MOST concepts related to the project is well defined in PPT	Knowledge of some concepts is defined in PPT	No knowledge of any of the concepts is presented.
Report	Reports must be well organized with the use case, class diagram, and activity diagram. The algorithm and outcome of the project are clearly defined.	The report is organized but not included in the use cases.	NOT well organized NOT submitted by the deadline
Mentoring	Students were engaged by a mentor in the lab classes and outside also.	A mentor engaged students in the lab classes.	Students are not helped at all.

### 3.3 Sudent performance assessment

Higher education needs common core standards as an integrated approach to deliver and assess the level of knowledge. In engineering, these core standards, at the abstract level, include critical thinking, problem-solving, decision making, communication, collaboration, and innovation.

**Figure 2:**
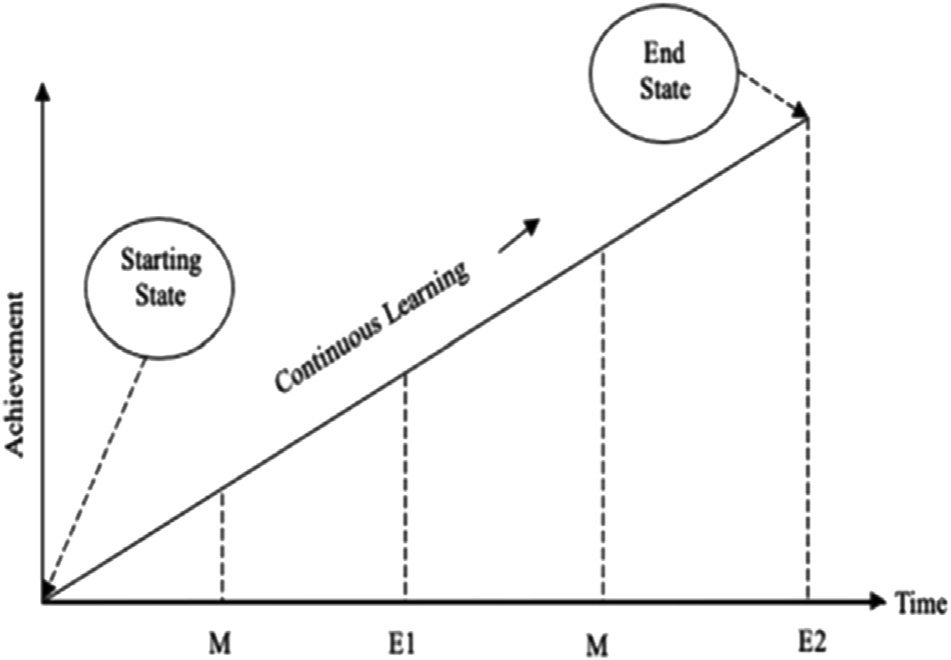
Proposed PBL framework.

There exists no single assessment method/technique that will guarantee the success of learning and assessment. Thus, it is essential to adopt an assessment system capable of assessing effective teaching and learning standards. The outlined process of the proposed framework is presented in Figure 2. The whole process of measuring attainment is divided into four parts, comprising of four evaluations divided into two mid evaluations (M1, M2) and two principal evaluations (E1, E2) carried out progressively at regular intervals over one year for undergraduate (final year engineering) students. Out of these four, only final evaluations (E1 and E2) will be considered to measure attainment levels.

### 3.4 Fuzzy inference method to calculate PAP's corresponding to CAP's

**Figure 3:**
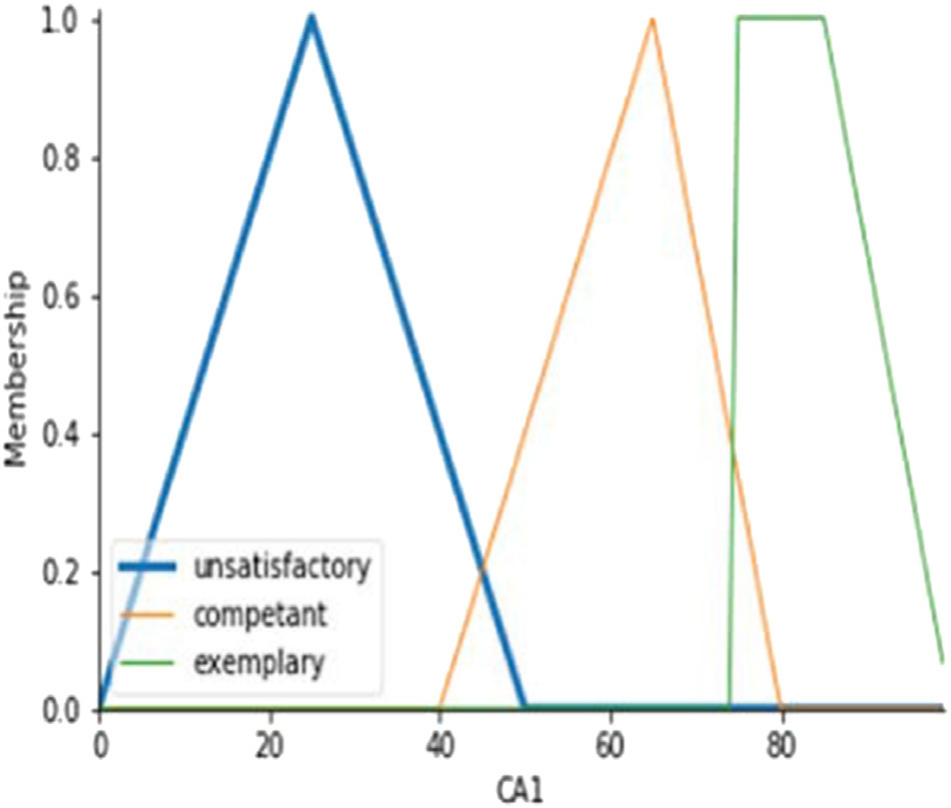
The Membership function for input variable (CAP1).

Mamdani Inference Method [13] is used to evaluate the PAPs corresponding to CAP's. The membership functions for these fuzzy variables are defined in Figure 3 for CAP1. The following are the steps for the Mamdani approach.

Step 1 Define the membership function for the input variable and the output variable.

The fuzzy variables exemplary, competent, and unsatisfactory are defined for the input variable course attainment parameter (CAP) using (1), (2), (3). There are three CAPs: CAP1, CAP2, CAP3. The membership function for each CAP is defined as:

(1) $$\mu_{unsatisfactory}=\left\{ \begin{array}{l} \frac{x-0}{25-0}\;;\;0<x\leq 25\;\\ \frac{50-x}{50-25}\;;\;25<x<50\\ 0\;;\;\text{otherwise} \end{array}\right.$$

(2) $${\text{μ}}_{\text{Competent}}=\left\{ \begin{array}{l} \frac{x-40}{65-40}\;;\;40<x\leq 65\\ \frac{80-\text{x}}{80-65}\;;\;65<x<80\;\\ 0\;;\;\text{otherwise} \end{array}\right.$$

(3) $$\mu_{exemplary}=\left\{ \begin{array}{l} 1;\;75<x\leq 85\\ \frac{100-x}{100-85}\;;\;85\leq x\leq 100\\ 0;\;\text{otherwise} \end{array}\right.$$

**Figure 4:**
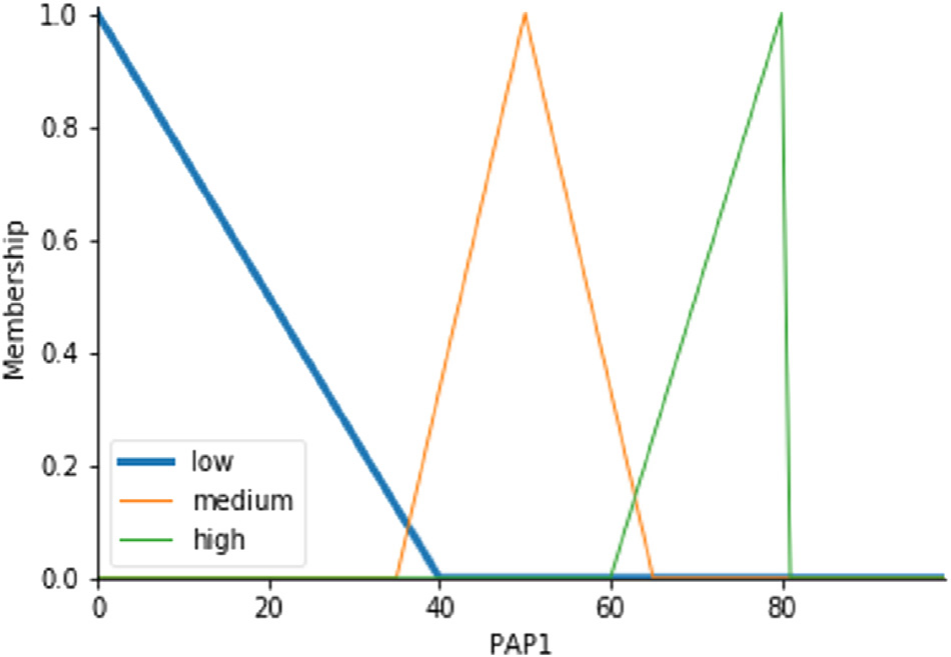
The Membership function for output variable (PAP1).

The project attainment parameter is considered as an output variable. The fuzzy variable of this output variable is low, medium, and high, measured on a scale of 80 using (4), (5), (6). Figure 4 shows the diagram for the membership function for the output variable project attainment parameter.

(4) $$\mu_{low}=\left\{ \begin{array}{l} \frac{40-x}{40-0}\;;\;0\leq x\leq 40\;\\ 0\;;\;\text{otherwise} \end{array}\right.$$

(5) $$\mu_{medium}=\left\{ \begin{array}{l} \frac{x-35}{50-35}\;;\;35\leq x\leq 50\\ \frac{65-x}{65-50}\;;\;50\leq x\leq 65\\ 0\;;\;\text{otherwise} \end{array}\right.$$

(6) $$\mu_{high}=\left\{ \begin{array}{l} \frac{x-60}{80-60}\;60\leq x\leq 80\\ 0\;,\;\text{otherwise} \end{array}\right.$$

Step 2 Define the rules.

Rules have been defined for each PAPs corresponding to CAPs. These are summarized in Table 3 below:

**Table 3:** Rules.

	Rules
For PAP 1	• R1: If CAP1 is unsatisfactory or CAP2 is exemplary, then PAP1 is medium. • R2: If CAP1 is exemplary, then PAP1 is high. • R3: If CAP1 is competent or CAP2 is exemplary, or CAP3 is unsatisfactory, then PAP1 is medium.
For PAP 2	• R1: If CAP1 is unsatisfactory or CAP2 is exemplary, then PAP2 is medium. • R2: If CAP1 is exemplary, then PAP2 is high. • R3: If CAP1 is competent or CAP2 is exemplary, then PAP2 is high. • R4: If CAP1 is competent or CAP2 is exemplary, or CAP3 is unsatisfactory, then PAP2 is medium.
For PAP 3	• R1: If CAP1 is unsatisfactory or CAP2 is exemplary then PAP3 is medium. • R2: If CAP1 is exemplary, CAP2 is exemplary or CAP3 is exemplary, then PAP3 is high. • R3: If CAP1 is competent or CAP2 is exemplary, then PAP2 is high. • R4: If CAP1 is competent or CAP2 is exemplary, or CAP3 is unsatisfactory, then PAP3 is medium.
For PAP 4	• R1: If CAP1 is unsatisfactory or CAP2 is competitive, not then PAP4 is low. • R2: If CAP3 is exemplary, then PAP4 is high. • R3: If CAP1 is competent or CAP2 is exemplary, then PAP4 is medium. • R4: If CAP1 is competent or CAP2 is exemplary, or CAP3 is unsatisfactory, then PAP4 is medium.
For PAP 5	• R1: If CAP1 is unsatisfactory or CAP2 is competent, then PAP5 is medium. • R2: If CAP3 is exemplary, then PAP5 is high. • R3: If CAP1 is competent or CAP2 is exemplary, or CA3 is exemplary, then PAP5 is medium. • R4: If CAP1 is exemplary or CAP2 is unsatisfactory, or CAP3 is exemplary, then PAP5 is medium.
For PAP 6	• R1: If CAP1 is unsatisfactory or CAP2 is competent, then PAP6 is medium. • R2: If CAP3 is exemplary, then PAP6 is high. • R3: If CAP1 is competent or CAP2 is exemplary, or CAP3 is unsatisfactory, then PAP6 is medium. • R4: If CAP1 is competent or CAP2 is exemplary, or CAP3 is exemplary, then PAP6 is high. • R5: If CAP1 is exemplary or CAP2 is unsatisfactory, or CAP3 is exemplary, then PAP6 is low.
For PAP 7	• R1: If CAP1 is unsatisfactory or CAP2 is competent, then PAP7 is low. • R2: If CAP1 is competent or CAP2 is exemplary, or CAP3 is unsatisfactory, then PAP7 is medium. • R3: If CAP1 is competent or CAP2 is exemplary, or CAP3 is unsatisfactory, then PAP7 is low. • R4: If CAP1 is competent or CAP2 is exemplary, or CAP3 is exemplary, then PAP7 is high.
For PAP 8	• R1: If CAP1 is competent or CAP2 is unsatisfactory, or CAP3 is unsatisfactory, then PAP8 is low. • R2: If CAP1 is competent or CAP2 is exemplary, or CAP3 is exemplary, then PAP8 is medium. • R3: If CAP1 is competent or CAP2 is exemplary, or CAP3 is exemplary, then PAP8 is medium.
For PAP 9	• R1: If CAP1 is competent or CAP2 is unsatisfactory, or CAP3 is unsatisfactory, then PAP9 is low. • R2: If CAP1 is competent or CAP2 is exemplary, or CAP3 is exemplary, then PAP9 is high. • R3: If CAP1 is unsatisfactory or CAP2 is competent then PAP9 is medium.
For PAP 10	• R1: If CAP1 is competent or CAP2 is unsatisfactory, or CAP3 is unsatisfactory, then PAP10 is low. • R2: If CAP1 is competent or CAP2 is exemplary, or CAP3 is exemplary, then PAP10 is medium. • R3: If CAP3 is exemplary, then PAP10 is medium.
For PAP 11	• R1: If CAP1 is competent or CAP2 is unsatisfactory, or CAP3 is unsatisfactory, then PAP11 is low. • R2: If CAP1 is competent or CAP2 is exemplary, or CAP3 is exemplary, then PAP11 is medium. • R3: If CAP3 is exemplary, then PAP11 is medium.
For PAP 12	• R1: If CAP1 is competent, or CAP2 is unsatisfactory, or CAP3 is unsatisfactory, then PAP12 is low. • R2: If CAP1 is competent, or CAP2 is exemplary, or CAP3 is exemplary, then PAP12 is medium. • R3: If CAP3 is exemplary, then PAP12 is medium.

All the consequent rules membership functions are combined using the aggregation function into a single fuzzy set. The input for the defuzzification process is the aggregate output fuzzy set, and the output is a single number. Defuzzified values of the fuzzy reasoning are derived based on the center of gravity –COG, the Mamdani-inference method. The next section discusses the results achieved from the above hypothesis. The equation of defuzzification is given (7) below:

(7) $$COA\left(A\right)=\;\frac{\sum \mu_{A}\left(x\right)\times x}{\sum \mu_{A}\left(x\right)}$$

## 4 Data collection

In this article, the CAP's assessment of professional college final year project students is taken into consideration. Table 4 presents the details of the participants:

**Table 4:** Participants details.

Characteristics		Number/Level
Age	Male	Between 21-22
	Female	Between 21-22
Gender	Male	442
	Female	173
Year of Study	Final Year of Engineering	615
Skills	Developed Minor Projects in two consecutive Semester	
Motivation	As aspiring for placement	High

Besides, each student's performance on each evaluation of the project has been considered based on defined 4Ç's corresponding to CAP's as shown in Table 5.

**Table 5:** Marks distribution of project.

Distribution of Marks according to 4C's (in percentage)				
	Communicate (CA3)	Cooperative (CA3)	Creative Thinking (CA1)	Critical Thinking (CA2)
E1	9	24.5	22.2	44.4
E2	39.5		23.2	37.2

E1 = Evaluation 1 and E2 = Evaluation 2.

Table 4 shows that in evaluation-1 (E1), the students were evaluated for communication is 9%, for cooperation, 24.5%, for creative thinking, 22.2%, and for critical thinking, 44.4%. Likewise, the marks are distributed for evaluation-2 (E2), focused on communication skills, creative thinking, and critical thinking. The performance of the students accordingly is shown in Table 6.

**Table 6:** Performance of the students according to 4C's.

Student performance according to 4Ç's (in percentage)				
Scores versus 4C's		Creative Thinking (CA1)	Critical Thinking (CA2)	Communicate and Cooperative (CA3)
E1	>50	97.4	76.6	86.2
	between 50 to 70	43.5	35.7	54.9
	between 70 to90	33.6	30.5	23.7
	>90	8	1	0.03
E2	>50	92.2	89.6	90.4
	between 50 to 70	44.5	37.3	38.6
	between 70 to90	39.8	43.9	47.9
	>90	2	2	1

E1 = Evaluation 1 and E2 = Evaluation 2.

It can be seen that students who have scored >90 are less percentage of students who performed well according to 4C's.

## 5 Empirical validation

Comprehensive empirical validation of the proposed method (refer to Table 7, Appendix A) is carried out as a controlled experiment with real subjects and data on 615 students. Learning analytics (as discussed in Table 5 and Table 6) is used to collect, analyze, measure, and report investigating data to understand the impact of the proposed assessment measure's success. The proposed approach's effectiveness is depicted by a statistical analysis performed on the result evaluation and performance of the undergraduate students and answers the research questions identified in section 1.

### 5.1 Analysis of project scores as course attainment parameters CAP1, CAP2, and CAP3 according to course learning outcome (RQ1)

The statistical analysis results investigated by analyzing each student's scores are summarized in Tables 8 and 9 presents the summary of Evaluation-1 results, whereas Table 9 represents the summary of results Evaluation-2 and answer the claim for the following null hypothesis (H0) and the alternative hypothesis (H1):

• Null Hypothesis (H0): For a given numerical data having a mean value less than or equal to 5.0 indicates a less understanding of the course objective. i.e., H, 0: sample mean 5.0 (no correlation)
• The alternative hypothesis (H1): For a given numerical data having a mean value larger than 5.0, indicates the understanding of course objectives, i.e., H1: sample mean >5.0 (correlation)

**Table 8:** Statistical analysis results of Evaluation-1.

	CAP 1	CAP 2	CAP 3
Mean	4.28	7.10	5.51
Variance	0.820	4.675	1.292
p-value	<0.001	<0.001

**Table 9:** Statistical analysis results of evaluation -2.

	CAP 1	CAP 2	CAP 3
Mean	6.88	10.75	11.24
Variance	2.019	5.591	4.972
p-value	<0.001	0.00018	

Table 9 presents the summary of Evaluation-2 results to answer the following null hypothesis (H0) and the alternative hypothesis (H1):

• The null hypothesis (H0) here is that, for a given question, the numerical data collected have a mean value less than or equal to 10.0, indicating no understanding to attain the parameter of CAPs improve with the help of the evaluation Panel.
• The alternative hypothesis (H1) is that the mean value is larger than 10.0, indicating an understanding and correlation between Bloom's taxonomy. H0: sample mean 10.0 (no correlation) H1: sample mean >10.0 (correlation).

The p-value is lower than 0.05, confirming a correlation for all the course attainment parameters with the scores. Therefore, the hypothesis of correlation should not be rejected for all course attainment parameters. Table 9 assumes a mean value larger than 10; on a scale of 1–20, indicating an improvement in the students' performance score taken by the evaluation panel.

### 5.2 Analysis of CAP1, CAP2, CAP3, and performance of students (RQ2)

Table 10 and Table 11 presents the linear relationship (correlation) between the variable of evaluation-1 and evaluation-2, respectively, for the following null hypothesis (H0) and the alternative hypothesis (H1):

• The null hypothesis (H0) here is that, for a given question, the numerical data collected has a mean value of score less than or equal to 5.0, indicating performance is poor to attain the parameter of CAPs.
• The alternative hypothesis (H1) is that the mean value of scores is more extensive than 5.0, indicating better performance to attain the parameter of CAPs.

**Table 10:** Correlation between the variables for Evaluation-1.

	CAP 1	CAP 2	CAP 3
CAP 1	1		
CAP 2	0.406352	1	
CAP 3	0.341504	0.27224805	1

**Table 11:** Correlation between the variables for Evaluation -2.

	CAP 1	CAP 2	CAP 3
CAP 1	1		
CAP 2	0.514697	1	
CAP 3	0.512833	0.650026	1

The positive correlation between the course attainment parameters confirms that if students have knowledge captured by parameters of CAP1, then the student has knowledge of other parameters and belonging to different CAPs. Table 12 and Table 13 present the descriptive statistics of each CAPs for Evaluation-1 and Evaluation-2.

**Table 12:** Project scores in Evaluation-1.

CAP1		CAP2		CAP3	
Mean	4.282	Mean	7.104	Mean	5.518
SD	0.905	SD	2.162	SD	1.1367
Max	6	Max	11	Max	11

SD = Standard Deviation, Max = Maximum.

**Table 13:** Project scores in Evaluation-2.

CAP1		CAP2		CAP3	
Mean	6.877	Mean	10.749	Mean	11.241
SD	1.421	SD	2.365	SD	2.230
Max	10	Max	15	Max	16

SD = Standard Deviation, Max = Maximum.

Table 12 shows the mean value of CAP1 is 4.282, which is less than the value 5, which explains the students' poor performance compared to other course attainment parameter's in Evaluation-1. In Evaluation-2 (refer to Table 13), the mean value of project scores is greater than 5, indicating the student's better performance in each CAPs.

### 5.3 Analysis of fuzzy scores of CAP1, CAP2, CAP3 corresponding to PAP1, PAP2, PAP3 … PAP12 (RQ3)

• Experts mapped course attainment parameters with program attainment parameters at a scale of 1–3, where 1 represents the slight, 2 for moderate, and 3 for substantial [36]. It is difficult for the teachers to map each CAP to each PAP at the right scale. Table 14 (Appendix A) shows the descriptive statistics of the proposed methodology of defuzzification of PAPs values. The high confidence level is above 95%, and it can be assigned as 3, whereas a confident level between 55 to 95 can be given as 2, and below 55% can be given as 1. Thus, the proposed Mamdani approach may help the teachers assign the PAPs values corresponding to the students' CAP scores. It will reduce the challenge to map the PAP's value corresponding to CAP parameters. The limitation of this approach is that it is perceived based on the expert rule where the rules are defined on assumptions that may or may not be accurate. Moreover, proposed approach can't learn pattern recognition as compared to machine learning and neural network type pattern recognition.
• The proposed method can be extended to find out the clusters between the courses and the program objective using machine learning algorithm, Neural network, multiple attribute decision making by using Archimedean norm operations [38]. Future work proposed Pilot testing of the tool for different subjects of outcome-based learning. The next section discusses the result validation of the inference results using the machine learning algorithms.

## 6 Result validation

This section discusses the machine learning algorithms to validate the inference results discussed in the section above. After defuzzification, the rule of aggregation of PAP's value is applied to determine the value of output variable whether the PAP corresponding to CAP is low, medium, high. In contrast, low represent 1, medium represents 2, and high represents 3, respectively. Various machine learning algorithms such as logistic regression, random forest, and naïve-based algorithms validate the classification of these PAP's values. The ratio of the training and testing data set is 70 and 30, respectively. There are other classification machine learning algorithms such as K-NN, A-NN for validation, can consider for future work. The proposed algorithm's result is measured in terms of recall, precision, and f1-measure, as shown in Table 15 (Appendix A). Table 15 shows that the random forest algorithm is achieved the highest accuracy, i.e., 98%, among others of the results obtained from the Mamdani inference system. The other machine learning classification algorithm also produced significant results. Hence, the proposed approach can be used to allocate the program attainment parameter corresponding to the course attainment parameter.

### 6.1 PAP attainment by a student

**Figure 5:**
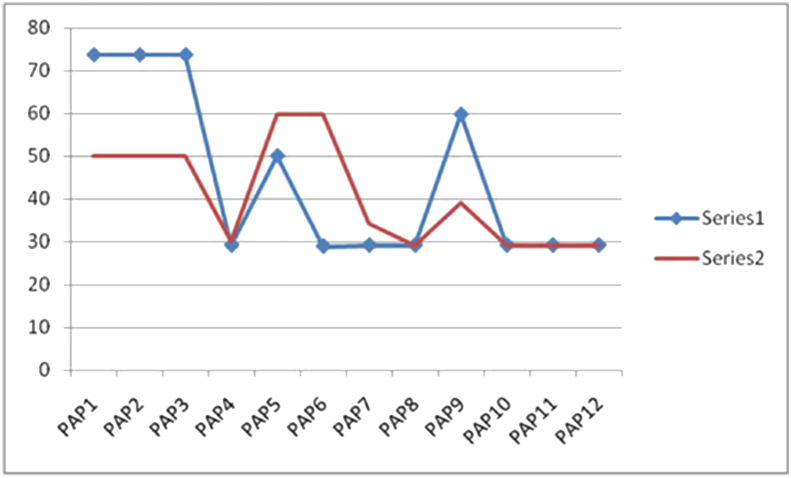
PAP attainment by two students.

The Jupiter platform, which was built in Python 3.7, has been used to apply the concept of outcome-based education using a fuzzy approach. A method for mapping the CAPs to the PAPs has been proposed. Furthermore, this method can be used to evaluate the system's attainment levels. Individual students' PAP attainment levels are indicated in Figure 5. It may aid in achieving the institution's goal, vision, and purpose with greater accuracy and precision.

## 7 Conclusions

This paper proposes a project-based learning framework to attain the competencies required by analyzing the effects of PBL implementation for engineering undergraduate-level courses. The fuzzy logic method, Mamdani Inference Method, evaluates the PAPs corresponding to CAPs to handle the vague and uncertain correlation mapping. The course attainment parameter is achieved from the student's project scores based on the proposed rubric. To the best of our knowledge, the literature lacks studies defining a well-defined mathematical tool to map these CAPs to PAPs. Results show that the undergraduate students' performance explores the fulfillment of learning outcomes/skills required to include creative and critical thinking, collaboration, communication, and creativity, with realistic constraints and standards for assessment in outcome-based educational environments.

Specifically, three research questions were analyzed (a) is there any effect of course learning outcomes on student performance? (b) does the student performance affect the course attainment parameter? and (c) can there be a mathematical tool to establish the relation between the course attainment parameter (CAP1, CAP2, CAP3) and program attainment parameter (PAP1, PAP2,…PAP12).

Results of experimentation to measure the proposed approach's effectiveness is depicted by statistical analysis and machine learning algorithms by achieving an accuracy of 98%. The authors have tried to investigate the impact of knowledge gained through project-based learning on (a) course attainment and (b) on overall engineering program attainment to promote deeper learning of 21st-century skills that students need to succeed in today's knowledge-based economy. The authors also believe that implementing PBL for undergraduate levels is more useful for training and making students aware of always working towards a sustainable future.

**Theoretical implication:** The paper makes two contributions to the body of knowledge. First, by expanding the corpus of knowledge and, second, by introducing new method to foster assessment in a highly dynamic environment that differ from what has previously been done. To the best of the author's knowledge, to date, there is no defined mathematical tool to map CAPs to PAPs. Thus, this paper proposes assessment pedagogy to evaluate the PAPs corresponding to CAPs to handle the vague correlation mapping using fuzzy logic.

**Practical implication:** First, instructors can apply the methodology in their courses for assessment in their own working environment, and with their internal competencies. The empirical evidence might easily be incorporated into almost every course at institute as well as university level.

One the limitation of this study is that Fuzzy theory cannot handle the imprecise data. If the data is imprecise then the proposed system will not infer the right relation between the CO and PAP mapping. In this study the participants were final-year computer science students. It's acceptable to believe that the findings of the study are representative of this population. Any application of the findings to the other set of students or courses must be considered with care.

Furthermore, the authors would like to study how other individual factors such as gender, prior knowledge, and experience affect students' competency in future research.

## Declarations

### Author contribution statement

Mukta Goyal, Chetna Gupta, Varun Gupta: Conceived and designed the experiments; Performed the experiments; Analyzed and interpreted the data; Contributed reagents, materials, analysis tools or data; Wrote the paper.

### Funding statement

This research did not receive any specific grant from funding agencies in the public, commercial, or not-for-profit sectors.

### Data availability statement

Data associated with this study has been deposited at Harvard Dataverse at https://doi.org/10.7910/DVN/7DRBDO

### Declaration of interests statement

The authors declare no conflict of interest.

### Additional information

No additional information is available for this paper.

## Figures and Tables

**Table 7:** Method of Empirical Validation

	Hypothesis 1	Hypothesis 2	Hypothesis 3
Goal	Analyze the score represent the attainment parameter according to Bloom Taxonomy	Analyze the relationship between course attainment parameters and Student Scores	Analyze the relationship between course attainment parameters with program attainment parameters.
Independent Variables	Scores in each CAP	Scores in each CAP	Total Course attainment value
Dependent Variables and measures	Total percentage in each CAP	Total Percentage in each CAP	Program attainment parameter
Empirical Study Approach	Simulation with data set of 615 using t-test	Simulation with data set of 615 using correlation and descriptive statistics	Mamdani Inference System with data set of size 615 analysis using t-test
Result Validation			Logistic Regression, Linear SVM, Random Forest, and Naïve Bayes validate the Fuzzy Results.

**Table 14:** Descriptive statistics of PAP's corresponding to each Course attainment

	PAP1	PAP2	PAP3	PAP4	PAP5	PAP6	PAP7	PAP8	PAP9	PAP10	PAP11	PAP12
Mean	56.674	57.4044	58.4495	38.6777	57.5777	44.6256	35.8803	31.4709	46.3308	33.101	33.101	33.101
Standard Deviation	9.28695	9.6761	8.84753	9.3277	3.73623	12.2969	5.35636	5.704	12.7859	7.19816	7.19816	7.19816
Minimum	50	50	50	16.539	50	13.3333	16.539	13.3333	18.432	13.3333	13.3333	13.3333
Maximum	73.6667	73.6667	73.6667	59.7051	59.7051	59.7051	56.0967	50	73.6667	59.7051	59.7051	59.7051
Conf Level (95%)	0.73724	0.76813	0.70235	0.74047	0.2966	0.97618	0.42521	0.45281	1.015	0.57142	0.57142	0.57142

Conf Level = . Confidence level.

**Table 15:** Validation of Algorithm using Machine Learning.

Score	Logistic Regression			Linear SVM			Random Forest			Naïve Bayes		
	precision	recall	f1-score	precision	recall	f1-score	precision	recall	f1-score	precision	recall	f1-score
Low	0.93	0.97	0.95	0.91	0.97	0.94	1.00	1.00	1.00	1.00	1.00	1.00
Medium	0.86	0.73	0.79	0.96	0.56	0.71	0.95	0.98	0.96	0.76	0.78	0.77
High	0.91	0.95	0.93	0.83	0.97	0.9	0.99	0.97	0.98	0.88	0.87	0.88
	Accuracy = 0.91			Accuracy = 0.88			Accuracy = 0.98			Accuracy = 0.90		
